# Travelling light: white sharks (*Carcharodon carcharias*) rely on body lipid stores to power ocean-basin scale migration

**DOI:** 10.1098/rspb.2013.0836

**Published:** 2013-09-07

**Authors:** Gen Del Raye, Salvador J. Jorgensen, Kira Krumhansl, Juan M. Ezcurra, Barbara A. Block

**Affiliations:** 1 Hopkins Marine Station, Department of Biology, Stanford University, 120 Oceanview Boulevard, Pacific Grove, CA 93950, USA; 2 Department of Oceanography, University of Hawaii, 1000 Pope Road, Honolulu, HI 96822, USA; 3 Monterey Bay Aquarium, 886 Cannery Row, Monterey, CA 93950, USA; 4 Department of Oceanography, University of Dalhousie, Halifax, Nova Scotia, Canada

**Keywords:** white shark, migration, bioenergetics, buoyancy

## Abstract

Many species undertake long-distance annual migrations between foraging and reproductive areas. Such migrants depend on the efficient packaging, storage and utilization of energy to succeed. A diverse assemblage of organisms accomplishes this through the use of lipid reserves; yet, it remains unclear whether the migrations of elasmobranchs, which include the largest gill breathers on Earth, depend on such a mechanism. We examine depth records from pop-up satellite archival tags to discern changes in buoyancy as a proxy for energy storage in Eastern Pacific white sharks, and assess whether lipid depletion fuels long-distance (approx. 4000 km) migrations. We develop new algorithms to assess body condition, buoyancy and drift rate during drift dives and validate the techniques using a captive white shark. In the wild, we document a consistent increase in drift rate over the course of all migrations, indicating a decrease in buoyancy caused by the depletion of lipid reserves. These results comprise, to our knowledge, the first assessment of energy storage and budgeting in migrating sharks. The methods provide a basis for further insights into using electronic tags to reveal the energetic strategies of a wide range of elasmobranchs.

## 1. Introduction

Migration optimizes resource availability by allowing migrants to take advantage of seasonal changes in distant environments [1]. Such behaviours often necessitate long sojourns through suboptimal habitat. Thus, migrants commonly cease to seek resources during their journey, supplanting localized foraging activity with directed movement and relying instead upon stored energy and nutrients to supply the cost of travel [2]. In avian long-distance migrants, for example, the accumulation of stored energy can cause pre-migration body mass to easily surpass twice the normal body weight [3]. Similarly, European eels (*Anguilla anguilla*) require a minimum threshold of body fat content before they embark on their prolonged spawning migrations to the Sargasso Sea [4]. Lipids are usually the preferred energy storage molecules in migrants because of their high-energy density and because they can be synthesized from any type of ingested food [5].

Recent studies indicate that white sharks, *Carcharadon carcharias*, are prodigious migrants. Satellite tagging has revealed extensive movement patterns [6,7] that involve large-scale trans-oceanic movements coupled with fidelity to coastal foraging sites [6–9]. In the California Current ecosystem, white sharks have been shown to migrate seasonally between highly productive inshore waters, where pinnipeds are a principal food item [7], and oligotrophic offshore waters, where prey is thought to be scarce. Although these movements have been well documented using the current generation of electronic tags as well as isotopic data [10], the physiology and energetics of these migrations remain challenging to resolve with current technologies.

As in other long-distance migrants, stored energy rather than locally obtained resources could be a major factor in white shark migration strategy. All elasmobranchs possess highly developed liver lipid stores that may help to fuel migration behaviours. As the single largest visceral organ, a white shark's liver, for example, can account for 28% of adult body weight [11] of which 90% by volume may constitute high-energy lipids [12]. High levels of ketone oxidation in the muscles of pelagic sharks indicate that hepatic lipids are an important fuel for locomotion [13]. Furthermore, the consumption of lipid stores equivalent to just one large meal of whale blubber may be sufficient by one estimate [14] to sustain a white shark's metabolism for one and a half months, enough to power an entire migratory transit.

We hypothesize that metabolism of liver lipid stores during prolonged migration may be detectable from changes in an animal's buoyancy. A 456 kg white shark liver [15] containing 400 l of oil and storing approximately 2 million kcal of energy [16] for example, may provide 50 kg of buoyant lift—nearly enough to neutralize the weight of the shark in water. The buoyancy of lipid stores was first demonstrated to exert a strong effect on dive characteristics in northern elephant seals (*Mirounga angustrostris*) [17–19]. The effects were most notable during drift dives—gliding periods where buoyancy replaces active propulsion as the primary locomotive force [20]. Furthermore, ‘drift rate’ (the vertical component of terminal velocity during drift dives) was a sensitive indicator of both relative buoyancy and lipid storage when compared with both direct measurements with isotopically labelled water [18] and to common foraging indices, such as track linearity [21]. This novel technique developed in marine mammals, which permits discerning on board fuel utilization using electronic tags, presents a promising non-invasive method for monitoring energy storage in vertebrates in the wild; yet, so far its applicability has been restricted mostly to birds and marine mammals. White sharks, which undertake distant seasonal migrations and whose buoyancy is dominated by liver lipids [22], are ideal candidates for this approach, given the recent application of satellite tags.

The objectives of this study were to use time-series data collected on electronic tags and video monitoring of captive animals: (i) to validate the use of drift rate (recorded by the pressure time series on an electronic tag) as a proxy for buoyancy and lipid storage in white sharks, (ii) to track changes in drift rate during long-distance migratory transits, and (iii) to determine whether white sharks rely on body energy stores rather than opportunistic foraging to fuel their migrations. We accomplished this by developing a novel adaptation of the technique proposed by Biuw *et al.* [18] in which we used drift rate to monitor lipid storage both in wild sharks tracked with electronic tags and in a controlled aquarium environment. Our results suggest that, when recorded at sufficient intervals, electronic tagging data from sharks can be used to monitor liver lipid utilization during migrations.

## 2. Material and methods

### (a) Conceptual model

**Figure 1.**
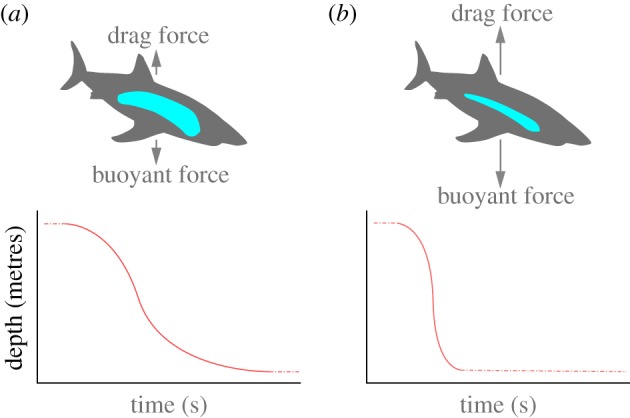
Conceptual model illustrating how body condition (lipid reserve) is expected to determine drift rate. (*a*) An increased liver (blue) mass (i.e. high-lipid reserves) results in a relative decrease in overall body density. In this scenario, lower drag forces are sufficient to reach terminal velocity during drift dives, yielding a slower drift rate (red line). (*b*) As lipid stores become depleted, the smaller liver mass results in an increase in overall body density leading to a faster drift rate.

Our investigation was guided by the following conceptual framework: during the summer and autumn, we hypothesize that foraging success of white sharks in the California Current ecosystem is manifest as a build-up of liver lipids. These lipids are generated from feeding on a rich diet of pinnipeds and have a strong positive effect on buoyancy (figure 1). In the winter and spring, the sharks undertake long migrations spanning thousands of kilometres from productive neritic habitats to offshore oligotrophic waters in the subtropical gyre. We hypothesize that they draw down their lipid stores during these transits, decreasing buoyancy.

Drift dives (defined as passive descent through the water column achieved without tail beats) offer an opportunity to monitor buoyancy. Many pelagic migrants use drift diving as an energy saving strategy [23], including white sharks [24]. We hypothesize that the drift rate (rate of vertical depth change) during these periods should reflect the shark's buoyant weight in a way that is subject to the following numerical description of a body in free fall through a medium of known density:2.1
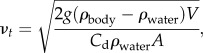
where *v_t_* is the drift speed determined by *ρ*_water_ the density of the medium, *ρ*_body_ the density of the body, *V* the volume of the body, *g* the acceleration of gravity, *C*_d_ the drag coefficient and *A* the wetted surface area of the body [18]. A global variance-based sensitivity analysis shows that *ρ*_water_ has a negligible effect on *v_t_* for the range of pressure, temperature and salinities through which the sharks migrate (see the electronic supplementary material, figure S1). Therefore, by controlling for *C*_d_ and assuming a constant *V* and *A*, we can derive that drift rate is proportional to the square root of body density, or in other words, the square of drift rate is inversely proportional to buoyancy.

We strove to minimize behavioural factors that could modify this proportionality. The most significant factors are active swimming by the shark and changes in pitch (the orientation of the shark's body with respect to the horizontal plane, i.e. angle of attack). Changing pitch violates the assumption of constant *C*_d_; however, *C*_d_ is robust to changes in pitch at shallow angles, and therefore, we strove to limit our analysis to dives with a small pitch (see the electronic supplementary material, figure S2). Active swimming violates the assumption of free fall; therefore, we sought to identify periods of passive locomotion (see the electronic supplementary material, figure S3). Deviation from true terminal velocity may be a problem at the beginning and end of a drift dive. However, a 1000 kg adult white shark is likely to reach a biologically realistic terminal velocity within a few seconds; therefore, this issue was circumvented by eliminating the first and last minute of each observed drift dive from consideration.

We made the implicit assumption in this analysis that the average density of liver lipids remains constant over time, and therefore that the buoyant weight of the shark is indicative of lipid volume. Sharks can however, modulate the composition of the oils in their liver to change their density [25]. We did not consider this to be important simply because we determined that this effect would be overwhelmed by the sheer volume of liver lipids involved. In the case of two white sharks chosen from a survey of just six individuals in the North Atlantic, for example [26], in the limiting case, the change in composition of liver lipids could account for only 12% of the difference in expected buoyant mass (see the electronic supplementary material, table S1).

### (b) Captive validation study

**Figure 2.**
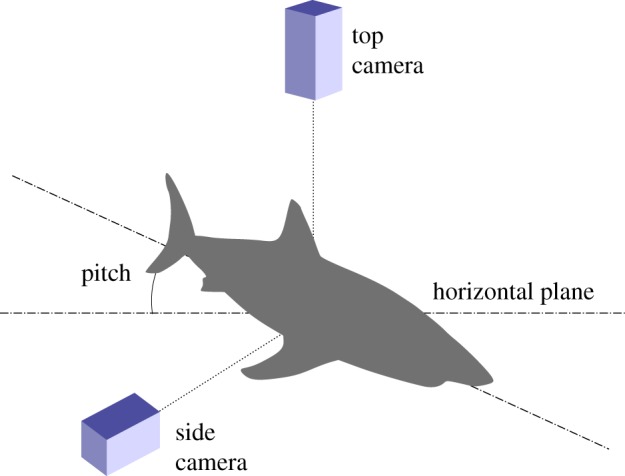
Captive shark experiment. Two fixed cameras outside the tank were used to track the movement of the shark across orthogonal planes. Pitch and the length-to-girth ratio were measured when the shark's body was parallel to the side camera. The occurrence of tail beats was detectable from the top camera. (Online version in colour.)

Our conceptual model was evaluated using direct observations acquired from a captive juvenile white shark. The shark was displayed in the Monterey Bay Aquarium's Outer Bay exhibit between 31 August 2006 and 16 January 2007 and had an initial length and body weight of 1.7 m and 47 kg, respectively, growing to a size of 2.0 m and 78 kg at the time of release. We used a dataset consisting of fixed position, orthogonal video images spanning a time period of 47 days (figure 2) to track the movement of the shark in three dimensions within the exhibit at 0.2 s resolution using the software package ImageJ (National Institutes of Health, Bethesda, MD, USA). Water temperatures (20°C) and light/dark cycles were kept constant between days. Length and girth were measured after capture and immediately before release, and growth rate was measured as the difference in weight between the start and end of captivity. We visually identified periods of passive drifting (defined as the absence of discernible tail beat for at least 5 s) and restricted our analysis to cases with a pitch of less than 10° (72% of dives). We compared the drift rate during these dives with an independent measure of body condition also obtained from the video images—the length-to-girth ratio of the shark. These procedures were designed to control for potentially confounding behavioural cues affecting drift rate, such as light, depth and temperature. Based on the observations of frequent and regular feeding events by the shark, our *a priori* hypothesis was that liver lipid stores and therefore body condition would increase over time and result in decreasing drift rate.

### (c) *In situ* application

Electronic tagging of free-swimming adult white sharks was carried out in the manner described in Weng *et al.* [7] and Jorgensen *et al.* [6]. Of 97 satellite pop-up archival tags (PATs) deployed on white sharks from central California (PAT v. 2.0, 3.0, 4.0 and Mk-10-PAT; Wildlife Computers, Redmond, WA, USA), we recovered time-series records from nine tags that had full archives of depth (pressure), temperature and light at intervals of less than or equal to 60 s. Light and temperature were used to estimate daily position using previously described methods of geolocation [27] and filtering via a state-space model [28,29]. Migration periods were clearly discernible in the data as monotonic increases or decreases in longitude that persisted for tens of days and linked longitudes of less than 125 W (the California Current) with longitudes of greater than 155 W (the Hawaiian Islands) [7]. We used standard movement indices such as track linearity [30] and daily travel speed to test whether the behaviours exhibited by the white sharks during these migrations were distinct from those observed near shore.

We gained additional insights into passive gliding during white shark swimming bouts using acceleration data from a multi-channel data logger [31] deployed on one adult coastal shark for a short period using previously described methods [32]. The data logger recorded acceleration in three axes at 5 Hz, as well as pressure and temperature over a period of 10 days. Overall dynamic body acceleration (ODBA), which is a measure of the magnitude of locomotory movements produced by an animal at a given time, was calculated using the procedures in Shepard *et al.* [33] using Python software (v. 2.4, Python Software Foundation 2007). The tail beat signature of the shark was clearly visible in the ODBA signal [32]. We defined drift dives in this analysis as periods where the ODBA revealed no discernible acceleration in any axis of the shark's body: i.e. when the ODBA fell below the 95% confidence threshold of the acceleration recorded by the data logger while stationary on a bench top.

A dive was defined as any series of monotonically increasing pressure readings. We selected drift dives from the time-depth archival datasets of the recovered PATs based on five factors: ‘centrality’ (the duration in time from a given point in a dive to its start or end), dive amplitude (the difference between the starting and the ending depth in a given dive), variance, dive length and migration length. Increasing centrality has been associated with decreased locomotion in diving animals [20,34]. We found that points in a dive sequence greater than 60 s from either the start or end of the dive (all points with a centrality greater than 60 s) were exclusively associated with passive drift diving (as defined by the 95% confidence threshold described earlier) in the accelerometer-equipped white shark (see the electronic supplementary material, figure S3). Similarly, dive amplitude is known to affect pitch in a wide range of animals [24,35,36] and correlated directly with an indicator of pitch derived from the accelerometer-equipped shark (see the electronic supplementary material, figure S4). As *C*_d_ is relatively robust to changes in body orientation at shallow angles of pitch (see the electronic supplementary material, figure S2), we used dive amplitude to limit our analysis to periods with putatively shallow pitch. Drift dives are also strongly associated with a low variability in movement velocity, because the rate of drifting should be reflective of the terminal velocity of a falling body. Therefore, we only considered periods with a maximum variability in sinking velocity of 1 m min^−1^. To enhance the statistical rigor of our drift rate estimates, we only considered dives with more than six data points (6 min of drifting), and used the average drift rate during these dives. Finally, to examine drift dives only during long-distance migrations, we focused our analyses on migration periods of duration exceeding one month (five transits and four individuals), as well as five periods of prolonged coastal residence that were used for comparison.

Dives that passed the above selection criteria were binned into 10-day intervals to eliminate any oscillations that occur at frequencies too high to be attributable to changes in buoyancy. This 10-day interval is similar to the spline smoothing window chosen by Biuw *et al.* [18] in their analysis of elephant seals. Based on the inverse-square relationship of equation (2.1), we calculated the slope of a least-squares linear fit of the squared drift rate over time to obtain the average rate of change in putative buoyancy for each period of interest. These average rates of buoyancy change were compared between migratory and coastal periods to test the null hypothesis that the positive slopes detected during migrations were statistically indistinguishable from coastal periods. This procedure treated each migration or coastal period as a single replicate.

## 3. Results and discussion

### (a) Drift rate is an indicator of body condition

**Figure 3.**
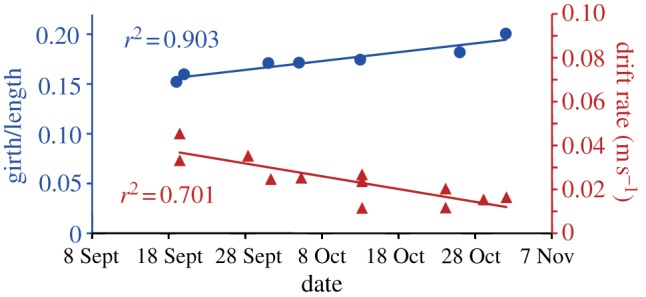
Drift rate versus body condition under captive conditions. Change in drift rate over 47 days for a captive juvenile white shark (red triangles) and change in girth-to-length ratio (blue circles) during the same period. Linear regressions are significant to *p* < 0.001.

The captive study on a juvenile white shark demonstrated that drift rate is a strong predictor of body condition. During the 47-day observation period of a captive white shark (initial length 1.7 m), there was a highly linear increase in body condition index (girth/length) (*n* = 7, *r*^2^ = 0.903, *F* = 46.0, *p* < 0.001) and a concomitant decrease in drift rate (*n* = 12, *r*^2^ = 0.701, *F* = 23.5, *p* < 0.001; figure 3). In captivity, the white shark was kept under optimal growth conditions at 20°C and fed to satiation with high-lipid food items (e.g. salmon). Ezcurra *et al.* [37] reported a mean growth rate (*n* = 4, including the individual described in this study) of 71.6 ± 8.2 kg yr^−1^ and 64.9 ± 8.5 cm yr^−1^ (mean ± s.e.) respectively, approximately twice the growth rate estimated from a Von Burtalanffy growth function for wild white sharks [37]. The steady decrease in drift rate correlates with an increase in body lipid content indicated by the rapidly improving body condition index assessed in captivity. There were no notable changes in pitch angle over time.

### (b) Movement patterns indicate that foraging is reduced during migration

**Figure 4.**
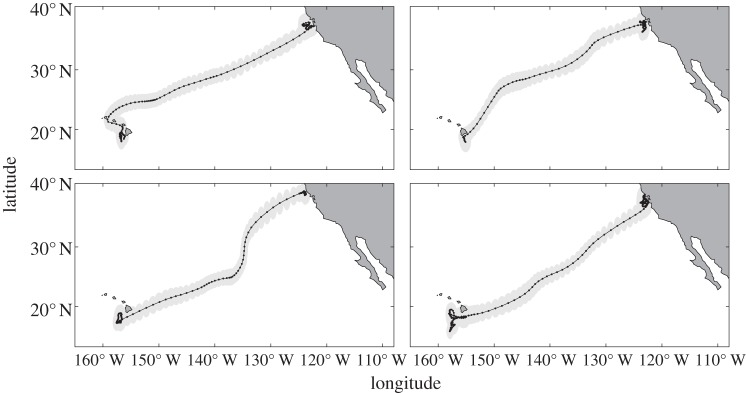
White shark migration trajectories. Median daily position estimates (points) from light/temperature geolocation fitted to a state-space movement model. Grey shading represents posterior distribution confidence limits (lower = 0.025, upper = 0.975, *n* = 2000 iterations). Highly linear movement patterns during migration suggest a predominance of directed travel over ARS behaviour during these periods.

Placement of electronic tags on adult white sharks permitted examination of the migration route and its linearity with geolocation observations. By examining the movement behaviour of four individuals, we determined that white sharks move rapidly and with a straighter course during transit than during other, non-migratory movement periods (figure 4). Mean daily travel distances during transiting migrations were 97.6 (*n* = 5; s.d. = 2.54) km d^−1^, necessitating a minimum swimming speed of 1.13 m s^−1^ to cover the straight-line distance between geolocation positions. This swimming speed is indistinguishable from previously reported observations of steady directional swimming in adult white sharks during coastal resident periods, which range between 0.89 and 1.34 m s^–1^ [38–40]. This indicates that steady directional swimming rather than local search behaviour may dominate movement during migration transits. High sustained swimming speeds have been shown to correlate closely with low levels of foraging success in other migratory predators [21]. Similarly, the mean track linearity (calculated as the ratio of the straight-line distance to the curvilinear distance) was 0.96 (*n* = 5 migrations; s.d. = 0.026), demonstrating that the migration trajectories were highly directional towards a distant destination and were markedly different from area-restricted search (ARS) behaviour such as is characteristic of foraging [39]. Track linearity during coastal periods, by contrast, showed a strong ARS imprint (mean = 0.27; s.d. = 0.061). These lines of evidence indicate a prevalence of relatively uninterrupted transiting and decreased frequency of foraging during migration.

### (c) Drift rate increases during migration

**Figure 5.**
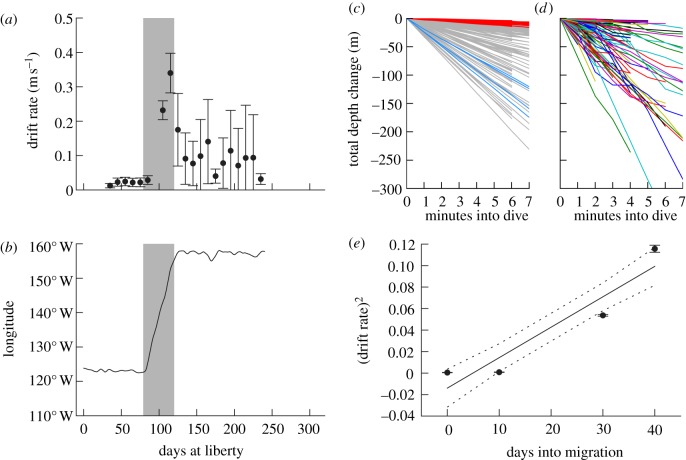
Representative track for a shark migrating between the California coastal zone and the Hawaiian Islands. (*a*) Drift rate over time compared with (*b*) longitude (as an indicator of migration). Longitudes 122°–124° W correspond to the California Coast and longitudes 155°–157° W to the Hawaiian Islands. Transiting migrations are indicated by grey shading. Error bars represent standard deviation. (*c*) Time series of depth change for dives used to estimate drift rate, with dives at the beginning of the migration in red, those at the end of the migration in blue and all remaining dives shown in grey. (*d*) A selection of 1000 dives selected at random from the records, showing a high variability in within-dive rate of depth change (nonlinear) compared with those selected for the calculation of drift rates. (*e*) Squared drift rate plotted against time. Points represent mean drift rates for 10 day intervals (*x*-axis value±5 days). Dotted lines represent 95% CIs of the least-squares fit.

**Figure 6.**
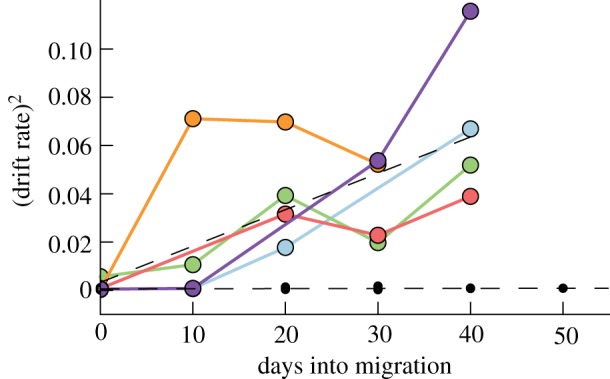
Drift rate profiles of migrant versus coastal sharks. Squared drift rate over time for five migrations (colour) versus five coastal records (black). Migrant sharks show a consistent rise in drift rate over time, implying a steady loss of lipid reserves, while coastal records show a buoyancy steady state. The single example of an eastward migration (green) shows a slope consistent with the other westward migrations. Black dotted lines show the average slope for each of the two groups. A two-tailed *t*-test revealed that the two samples were significantly different with a *p*-value < 0.005.

Drift rates measured during oscillatory dives in nine sharks (table 1) and displayed marked differences between migratory and non-migratory periods (figures 5 and 6). Migrants exhibited rapidly increasing drift rates with the slopes of the least-squares linear fit clustered tightly around a mean of 1.5 × 10^−3^ m s^−1^ d^−1^ (figure 6; *n* = 5 migrations) and were not significantly different between those transiting westward (California to Hawaii) and the single eastward migration (Hawaii to California). The increase in drift rates implies decreasing buoyancy, and therefore decreasing liver lipid reserves during migration. By contrast, sharks residing in coastal zones showed changes in drift rates over time clustering near or below zero (*n* = 5 tracks; mean = 5.0 × 10^−6^). A Student's *t*-test comparing all migrations against coastal tracks showed that the two sample groups were highly distinct (*p* = 1.5 × 10^−3^). This is consistent with sharks exclusively experiencing net lipid consumption during offshore migratory transits while the coastal periods appear to be characterized by a buoyancy steady state.

**Table 1.** Summary of drift dive data. (Both the duration of drift dives and the depth range are shown ±1 s.d.)

shark ID	drift dives (*n*)	duration (min±s.d.)	depth range (m±s.d.)
migration periods			
1	39	9.2 ± 2.2	63.8 ± 29.0
2	34	8.7 ± 1.1	63.0 ± 30.0
3	14	8.6 ± 0.9	88.3 ± 45.0
4	24	9.1 ± 1.1	70.4 ± 62.1
coastal periods			
1	8	8.0 ± 0.5	9.3 ± 2.1
2	46	8.0 ± 0.8	9.0 ± 2.2
3	46	8.6 ± 0.8	9.2 ± 2.7
4	10	8.5 ± 0.8	9.9 ± 2.8
5	602	7.2 ± 1.7	14.2 ± 9.1

A decrease in buoyancy in transiting sharks could be attributable to either a decline in lipid volume owing to metabolism of onboard lipid stores, or an increase in denser lean tissue (muscle mass) through exercise-induced conditioning. However, because the density of lean tissue is highly similar to the overall body density of elasmobranchs (difference of 0 and 0.007 compared with 0.015 and 0.022 g ml^−1^ for muscle and liver of male and female Port Jackson sharks, respectively [41]), muscle build-up due to continuous exercise is unlikely to become a controlling factor in buoyancy change. Therefore, the data are most indicative of a cessation or decline in foraging activity leading to a drawdown of lipid stores to power long-distance migration. Furthermore, as obligate ram ventilators, white sharks must swim continually during both migratory and resident phases. Average swimming speeds appear to be equivalent for both phases, therefore, there are unlikely to be large differences in lean tissue bulk during a phase of continuous migration.

Behavioural factors in swimming animals such as the pitch of the body (which alters the drag coefficient) or the lift generated by the body and pectoral fins are known to affect drift rate independently of buoyancy. Although our accelerometer data loggers only permitted us to obtain relatively short time series of pitch and tail beat in coastal sharks, we believe variability in these factors is unlikely to account for the patterns we observed in the buoyancy of migrants. Apart from the measures we used during data selection to standardize for swimming behaviour, pitch and lift-to-drag ratio are highly constrained in diving animals [24,42]. This applies particularly to lamnid sharks, which have limited mobility in their pectoral fins. Moreover, there is strong selection to optimize the efficiency of movement during long migratory transits given the high aerobic costs of locomotion, and this has been found to restrict variability in swimming behaviour across a wide range of animals for which such measurements have been made [43]. The power requirements of travel increase exponentially with higher pitch in whale sharks for example, leading pitch to approach minimal values in all dives [24]. Furthermore, using equation (2.1) (see §2), we calculate that a systematic decrease in drag coefficient in the vertical direction of 76% would be necessary to account for the observed trends in drift rate in migrating sharks (see the electronic supplementary material, equation S1). Such a bias would need to be exclusive to migratory rather than coastal swimming and would need to intensify with proximity to the destination independent of the direction (eastward or westward) of transit. This is highly unlikely given there is no readily available environmental or behavioural mechanism to support the existence of such a systematic bias over time. For these reasons, we feel that the observation of monotonic increases in drift rate in migrating sharks is most parsimoniously explained by depletion of body lipid stores. Newer accelerometer data loggers that are capable of measuring tail beat and pitch over prolonged periods (more than one month) may allow direct evaluation of some of these parameters in migrating white sharks in the future.

### (d) Conclusions and management implications

We present a new method for analysing satellite tag-derived depth time series to explore the diving behaviour, buoyancy and the role of lipids during shark migration. By validating this technique with direct observations, we have, to our knowledge, made the first attempt to study the energetics of white shark migration observed with satellite tags. Analysis of time-series data indicates that white sharks are initially buoyed up by ample lipid reserves early in migration but gradually lose this buoyancy as energy stores are consumed. We hypothesize that the capacity to store substantial energy in the liver is a key specialization for achieving ocean-basin scale migration. Future studies should focus on direct measurements of tail beat frequency during all phases of migration, and on acquisition of additional physiological measurements that will help discern in detail how onboard lipid stores are used during long-distance migration. Direct sampling and isotopic measurement of individual sharks pre- and post-migration may reveal which body lipids limit the ability of a shark to embark on these migrations.

While white sharks are the most widely protected elasmobranchs in the world [44], global populations are sparse and considered to be threatened or endangered [45,46]. Most targeted conservation measures have focused on limiting fishing-induced mortality, however, our results highlight migration as a potentially important seasonal energetic stress that may carry its own management implications. Increased energetic requirements both pre-migration (to build up adequate lipid stores) and post-migration (to recoup energetic losses) suggest a reliance on high prey availability during foraging seasons in the neritic coastal zones. Suboptimal prey availability during these periods through stock depletion or phenological mismatch could interact with ancillary stressors to compromise growth or reproductive success [47]. Our results could indicate that the intrinsic physiological demands of migration may be linked to the susceptibility of white sharks to potential disturbances or declines in prey availability. This underscores the importance of the period of intense foraging on pinnipeds that occurs seasonally along the coast of California. Furthermore, stable isotope data confirm that foraging does occur either offshore in the oceanic environment or near the Hawaiian Islands, but at a lower rate; approximately half of that during the coastal phase [10]. The principle prey species responsible for sustaining these white sharks in their offshore migration endpoints remains undetermined. Patterns of prey abundance in these regions warrant further research to determine whether they could be linked to the viability of white shark populations.

## Notes

This project was conducted under research permits from CDFG, MBNMS, GFNMS, NMFS, NPS and under Stanford University animal care protocol 10765.

## Data accessibility

Requests for data should be referred by email to the primary author. Every effort will be made to respond to requests in a timely manner.
